# Sex steroid hormone residues in milk and their potential risks for breast and prostate cancer

**DOI:** 10.3389/fnut.2024.1390379

**Published:** 2024-09-02

**Authors:** Pengyue Gao, Chengyi Li, Quan Gong, Lian Liu, Rui Qin, Jiao Liu

**Affiliations:** ^1^Hubei Provincial Key Laboratory for Protection and Application of Special Plant Germplasm in Wuling Area of China, College of Life Sciences, South-Central MinZu University, Wuhan, China; ^2^School of Basic Medicine, Yangtze University, Jingzhou, China

**Keywords:** sex steroid hormones, milk, breast cancer, prostate cancer, 17β-estradiol

## Abstract

Milk was a source of important nutrients for humans and was especially important for children and adolescents. The modern dairy animal production pattern had contributed to residual sex steroid hormones in milk. When this milk was consumed by humans, these hormones entered the body leading to hormonal disruptions and potentially increasing the risk of various types of cancers. This article reviewed the presence of residual sex steroid hormones in milk, their potential risks on human health, and their possible association with the incidence of breast and prostate cancer. The potential linkage between dairy consumption and these cancers were described in detail. The hormones present in dairy products could affect the development and progression of these types of cancer. Sex steroid hormones could interact with different signaling pathways, influencing carcinogenic cascades that could eventually lead to tumorigenesis. Given these potential health risks, the article suggested appropriate consumption of dairy products. This included being mindful not just of the amount of dairy consumed, but also the types of dairy products selected. More scientific exploration was needed, but this review provided valuable insights for health-conscious consumers and contributed to the ongoing discussion on dietary guidelines and human health.

## 1 Introduction

The “milk campaign” was a strategy adopted by many countries, promoting milk as a significant dietary element, especially among students, due to its rich content of nutrients (1–4). Global milk consumption per capita, at 76 kg/year, is generally increasing, although it varied significantly by region (5). For instance, in 2021, Denmark’s per capita milk consumption was 402.13 kg, while China’s per capita milk consumption was 34.22 kg. This variation was correlated with per capita income, as high-income countries tend to consume a larger amount of milk per capita (184.81 kg), middle-income countries consume between 65.72 to 82.71 kg of milk per capita, and low-income countries consume 30.94 kg of milk per capita (6). However, this essential source of nutrition was at risk of contamination with various substances (7–9), including sex steroid hormones, which might be harmful to consumers (10, 11). One significant hormone was 17β-estradiol (E2), a steroidal estrogen associated with endocrine disruption (12, 13). Its presence in milk increased the risk of hormonal disorders, reproductive and immune system abnormalities, and potentially cancer in consumers (14, 15). The consumption of milk has been linked with an elevated risk of various cancers, particularly breast cancer (BC) and prostate cancer (PCa), potentially attributed to the sex hormone content found in dairy products (16–18). Certain studies have indicated a positive correlation between milk consumption and the risk of developing BC (19). Chinese adults, with comparatively lower milk intake than the global population, have shown that dairy intake was positively linked to an elevated risk of liver cancer and female breast cancer (20). Moreover, the potential role of milk and the residual sex hormones present in milk in breast tumorigenesis has been investigated (21). The data suggested that increased consumption of dairy products might increase the risk of prostate cancer (22). Low fat milk intake was related to an increased risk of non-aggressive PCa, while whole milk intake was related to an increased risk of lethal PCa (23). This article reviewed the existence of sex hormone residues in milk, the relationship between milk consumption and the risk of breast and prostate cancer. The aim was to provide a comprehensive understanding of the benefits and potential risks associated with milk consumption, and recommendations for appropriate consumption to reduce potential cancer risk.

## 2 Scope and method

The Preferred Reporting Items for Systematic Reviews and Meta-Analyses (PRISMA) guidelines (24) were adhered to during the planning, execution, and reporting of this review. All literature up to December 2023 was searched in ‘PubMed’, ‘Embase’, ‘Scopus’ and ‘The Cochrane Library’ to identify relevant articles discussing the potential risk of breast and prostate cancer associated with hormone residues in milk. The terms ‘Breast Neoplasm’, ‘Prostatic Neoplasm’, ‘Gonadal Steroid Hormones’, ‘Estrogens’, ‘Androgen’, ‘17β-estradiol’, ‘Progesterone’, ‘Milk’, and ‘Dairy Products’ were employed as subject words for retrieval. To ensure comprehensive coverage and avoid overlooking relevant studies, references in the primary articles and related reviews were manually screened.

Databases including PubMed, Embase, Scopus, and The Cochrane Library were searched for all available records. The searches utilized search terms (‘Breast Neoplasm’ OR ‘Prostatic Neoplasm’) combined with (‘Gonadal Steroid Hormones’ OR ‘Estrogens’ OR ‘Androgen’ OR ‘17β-estradiol’ OR ‘Progesterone’) AND (‘Milk’ OR ‘Dairy Products’), specifically in the ‘title’ and ‘abstract’ fields. All free words were searched using the logical operator OR with the corresponding subject term for an inclusive search strategy. Additionally, reference lists of identified papers and review articles were manually searched to ensure no relevant papers were overlooked in our database searches. The objective was to identify as many relevant articles as possible related to our research aim.

**Figure 1:**
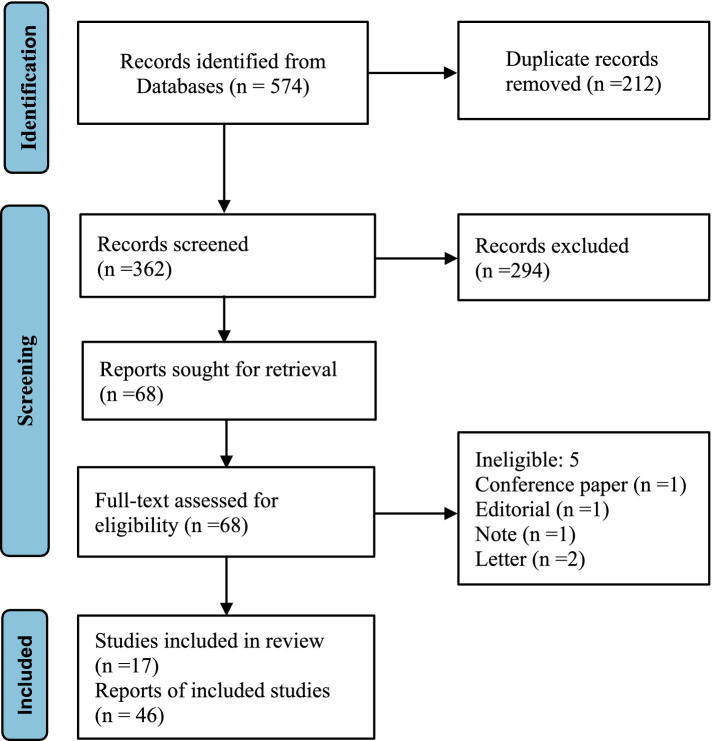
PRISMA flow diagram.

For inclusion, articles needed to meet the following criteria: The study explored the potential risk of breast or prostate cancer development associated with the presence of sex steroid hormones in milk. This could include case–control studies, prospective or retrospective cohort designs, studies on laboratory animals, or histopathology cultures. Exclusion criteria: (1) Studies with unavailable full-text access; (2) Duplicate cohorts or participants-the article should include the most recent or comprehensive information; (3) Studies published in languages other than English (Figure 1). Milk, being a natural nutrient, does not inherently contain hormones. This article exclusively focused on steroid sex hormones that could be present in the natural nutrient and their potential risk for breast and prostate cancer in humans.

## 3 Sex steroid hormone residues in milk and their potential risks for breast and prostate cancer

### 3.1 The presence and content of sex steroid hormones in milk

The production of sex hormones, such as those produced by the ovaries and placenta of animal, were a normal physiological occurrence. The ovaries were the primary sources of sex hormone production (25). Modern dairy livestock breeds tended to be in lactation most of their lives, even when pregnant, leading to an increased level of sex hormones in the milk they produced. The presence and variations in gonadal hormone content in milk generally mirrored the reproductive physiological traits of the dairy animal and generally aligned with levels found in the serum (26). This suggested that sex hormones regulated the reproduction of the dairy animal. Sex steroid hormones that occurred naturally in milk were secreted from internal glands and were carried over the blood-milk barrier, leading to their presence in raw milk (27, 28). Reproductive hormones were also used to stimulate estrous and maintain pregnancy in livestock production (29). The dynamic change of reproductive status caused the hormone content of milk derived from to change dynamically.

Improvements in animal breeding and farm management techniques have dramatically increased per animal milk production (30, 31). This increase could be changes in the lactating animal’s endocrine system, which subsequently impacted the residual hormone content in the milk (32). As sex hormones regulated reproduction, the hormone levels in a lactating animal’s blood often fluctuated based on the animal’s physiological reproductive conditions, leading to varying levels of hormonal residue in milk (Table 1). Milk from pregnant animals had the highest concentrations of estradiol, followed by milk from estrus animals, and the lowest levels were found in milk from non-reproductive animals (33). Milk composition also impacted hormone levels, with higher estradiol levels found in high-fat milks compared to low-fat milks (34) (Table 2). Differences in estrone (E1) and E2 content had also been observed between regular and organic milk, with organic milk found to have higher E1 and E2 content (35). Though further investigation was needed, some research suggested that reducing milk or dairy product intake, especially high-fat products, might help reduce cancer risks, particularly in individuals with a higher cancer risk to begin with (18, 36).

**Table 1:** Sex hormone levels in milk from cows with different physiological reproductive states (pg/ml).

Physiological reproduction conditions	E1(Estrone)	E2 (17β-estradiol)	Testosterone	4-Androstenedion	Progesterone	Detection method	References
Non-pregnant and non-estrus cows	88.0 ± 0.0		80.0 ± 19.0	109.0 ± 30.0	82.0 ± 31.0	LC–MS/MS	(35)
		87.9 ± 27.3				ELISA	(33)
		1.3 ± 0.2				RIA	(137)
Estrus cows		8.3 ± 1.2			15600.0 ± 1270.0	ELISA	(26)
		148.8 ± 7.3				ELISA	(33)
Pregnant cows	140.0 ± 95.0		103.0 ± 10.3	367.0 ± 40.6	824.0 ± 30.7	LC–MS/MS	(35)
Pregnant cows in the first trimester of gestation	7.9 ± 0.7	18.6 ± 0.2				LC–MS/MS	(30)
		0.9 ± 0.3				RIA	(137)
	0.6	0.3				RIA	(138)
		42.4–68.3				GC–MS	(139)
	452.0 ± 66.0	51.4 ± 2.7				LC–MS/MS	(30)
Pregnant cows in the second trimester of gestation		187.4 ± 27.3				ELISA	(33)
		3.0 ± 0.4				RIA	(137)
	7.9	0.9				RIA	(138)
		139.4 ± 11.8				GC–MS	(139)
Pregnant cows in the third trimester of gestation	1266.0 ± 38.0	51.0 ± 2.0				LC–MS/MS	(30)
	27.1	5.0				RIA	(138)
		1105.3 ± 78.5				GC–MS	(139)
	47.3 ± 4.2	36.1 ± 10.0				ELISA	(140)

LC–MS/MS, Liquid chromatography–tandem mass spectrometry; ELISA, enzyme-linked immunosorbent assay; GC–MS, Gas chromatography–mass spectrometry; RIA, radioimmunoassay.

**Table 2:** Sex hormones level in different types of milk (pg/ml).

Types	E1(Estrone)	E2 (17β-estradiol)	Testosterone	4-Androstenedion	Progesterone	Detection method	References
	7.9 ± 0.3	1.1 ± 0.05				RIA	(137)
Whole milk	159.0	6.0	<30.0	684.0	15486.0	LC–MS/MS	(141)
	130.0	<20.0	<10.0	210.0	9810.0	GC–MS	(142)
		282.2 ± 133.0			3100.0 ± 1830.0	ELISA	(36)
		288.0 ± 182.5			3690.0 ± 2380.0	ELISA	(36)
		318.0 ± 199.8			2740.0 ± 1850.0	ELISA	(36)
		443.4 ± 184.7			4770.0 ± 3120.0	ELISA	(36)
		198.6–918.3				ELISA	(143)
	129.9 ± 18.5	28.19 ± 5.3				LC–MS/MS	(144)
	152.8 ± 60.0	23.0 ± 12.6	78.1 ± 21.8	934.3 ± 270.9		LC–MS/MS	(145)
	187.0	13.5	9.0	876.0		LC–MS/MS	(146)
					1300.0–15500.0	LC–MS/MS	(147)
Half skim milk	138.7 ± 22.3	29.57 ± 5.3				LC–MS/MS	(144)
			51.3 ± 17.0	533.8 ± 101.4		LC–MS/MS	(145)
	141.1	13.0	9.9	421.9		LC–MS/MS	(146)
Low fat milk					4400.0–7000.0	LC–MS/MS	(147)
	2.9 ± 0.1	6.0 ± 0.3				RIA	(137)
Skim milk	129.2 ± 17.7	31.28 ± 3.8				LC–MS/MS	(144)
			31.8 ± 3.6	296.3 ± 86.5		LC–MS/MS	(145)
					1100.0–1400.0	LC–MS/MS	(147)
Cream	54.1 ± 2.8	6.0 ± 0.3				RIA	(137)
	260.0	<30.0	<30.0	1250.0	48600.0	GC–MS	(142)
Butter	118.9 ± 6.5	15.8 ± 1.2				RIA	(137)
					2400.0–3200.0	LC–MS/MS	(147)
Whole milk-Or	260.0 ± 28.3	61.5 ± 11.8				LC–MS/MS	(144)
					5900.0–14000.0	LC–MS/MS	(147)
Half skim milk-Or	240.5 ± 22.2	52.8 ± 6.1				LC–MS/MS	(144)
Skim milk-Or	175.4 ± 34.5	38.0 ± 6.9				LC–MS/MS	(144)
					510.0	LC–MS/MS	(147)

### 3.2 Sexual steroid hormone residues in milk and possible risk of breast cancer

BC accounted for a substantial proportion of total cancer cases and was the most common form of invasive cancer in the female population (37, 38). The incidence of BC was related to genetics. The high incidence of mutations in high-penetrance genes such as BRCA1 and BRCA2 among certain subpopulations has been linked to the elevated prevalence of BC (39). The incidence of BC was related to the living environment. It varied across different geographical regions, with notably higher rates observed in developed countries compared to developing countries. Furthermore, the death rate from BC was higher in regions with lower levels of economic development (38). The strong relationship between BC morbidity and standards of living, where the prevalence of BC risk factors was influenced by significant changes in lifestyle, sociocultural and built environment brought about by economic growth. The convergence of these influencing factors across countries reduced regional disparities in BC incidence (37). As a result, there was a complex interplay of socio-economic and lifestyle factors that determined BC risk.

Research on cancerogenesis has established the prevention strategies in the battle against cancer. Changes in the environment, lifestyle, and diet have been identified as pivotal factors in mitigating cancer risk (40). Dairy products could have both pro- and anti-carcinogenic effects. They contained a range of nutrients and bioactive compounds that could potentially influence cancer development. For instance, components such as vitamin D, and conjugated linoleic acid found in dairy have been associated with potential anti-carcinogenic effects due to their influence on cell processes, proliferation, and differentiation, which could inhibit tumor development (41–43). Conversely, certain components within dairy products, such as high levels of fats, and potential contaminants like pesticides, estrogen metabolites, and growth factors like IGF-1, might contribute to an increased risk of BC.

The relationship between milk intake and BC risk has been the subject of extensive research, both domestically and internationally. Some studies have suggested a positive correlation between milk consumption and the risk of developing breast cancer (43). The relationship between dairy intake and the risk of certain cancers, including female BC, has been investigated. Some studies suggested that higher dairy consumption might be associated with an increased risk of certain cancers (18). Chinese adults with relatively lower milk intake compared to the global population, but dairy intake has been positively associated with an increased risk of liver cancer and female BC (20). High consumption of fermented dairy products over an extended period has been linked to a potential reduction in the risk of ER- or progesterone receptor-negative (PR-) BC (44). These studies showed the complexities of the relationship between dairy consumption and cancer risk, particularly in the context of different cultures, dietary habits, and cancer subtypes. Furthermore, the potential role of milk and the residual sex hormones in milk in breast tumorigenesis has been explored (45). The presence of sex hormones in milk was potentially associated with breast cancer risk. Studies examining the role and mechanisms of residual sex hormones in milk have been conducted using cell cultures, animal models, and human breast cancer scientific models (46).

Meta-analysis of observational studies showed that the consumption of dairy products might have an overall reduction in BC risk in the female population. However, different types of dairy products might have varying effects on different subtypes of BC as well as the menopausal status of individuals (19). Studies have also shown a positive association between milk consumption and the risk of breast cancer, with this link observed to be independent of milk fat content (43). Specifically, consuming more than 750 mL of whole milk daily has been associated with an increased risk of BC (47). Moreover, milk intake has been positively linked to ER-BC risk and was strongly associated with ER+/PR+ tumors (43, 48). While the consumption of a relatively small amount of milk per day (158 mL) was associated with an increased risk of BC, the intake of cheese and yogurt was associated with a reduced risk of BC. Additionally, consistent consumption of non-fermented milk has been linked to an increased incidence of ER+/PR+ BC, particularly in women of normal weight (44). Conversely, there were negative correlations between the consumption of fermented dairy products and the risk of ER/PR cancers (44). Indeed, the association between milk consumption and breast cancer risk might be attributed to the composition of milk. Elevated serum levels of estradiol or testosterone (T) increased risk of breast cancer in postmenopausal women (49–51). But Mongolian women, whose dietary habits predominantly centered around the consumption of meat and dairy products, exhibited markedly elevated circulating levels of estradiol and progesterone before menopause compared to British women, despite the latter experiencing a higher incidence of breast cancer (52). High-hormone, including relatively high serum concentrations of oestradiol, T, prolactin, progesterone, and cortisol, was correlated with a five-fold increase in BC risk, independent of various potential confounding factors (53). Specifically, serum progesterone, have been associated with postmenopausal BC in various types of studies, including case–control, prospective, and cross-sectional studies (54–56). Progesterone played a role in breast cancer etiology by promoting the proliferation and differentiation of mammary epithelial cells (57). Moreover, increased serum level of insulin-like growth factor (IGF) 1 was also a risk factor for BC (58) because IGF-1 had a potential synergistic effect with estrogen in milk in breast cancer development (58, 59). The elevated estrogen levels in commercially available milk (Estrone, 436.2 pg./mL; 17β-Estradiol, 213.7 pg./mL; Estriol, 53.4 pg./mL) could potentially contribute to the promotion of DMBA-induced breast tumor development in rats, with a possible synergistic effect with other hormones (60). Hence, the hormone content present in milk could be a significant factor in the association between milk consumption and an elevated risk of breast cancer.

### 3.3 Sexual steroid hormone residues in milk and possible risk of prostate cancer

PCa was one of the most common malignancies affecting the male genitourinary system worldwide. The oncogenesis of PCa involved complex interactions between innate population susceptibility, acquired genetic mutations, and microenvironmental as well as macroenvironmental factors. When prostate cancer developed, genomic mutations could lead to atypical growth and division of the glandular tissue cells within the prostate, resulting in the formation of nodules or tumors (61, 62). The majority of prostate malignancies originated from the epithelial cells within the prostate gland. These cells underwent pathological changes that lead to the development of prostate cancer (63). During tumorigenesis, the complex signaling pathways of epithelial cells could be disrupted, leading to the progression from benign to malignant disease (64). In PCa, various mutations and defects impacted cell signaling pathways, hormone levels, and hormone receptors within the tissue. These alterations had the potential to disrupt the complex interplay between the stroma and epithelial cells within the prostate microenvironment.

The incidence and mortality rates of PCa varied significantly based on geographical and ethnic distributions, reflecting differences in varying degrees of genetic susceptibility to the disease (65, 66). First, genetic factors played a significant role in the risk of developing prostate cancer, the incidence of hereditary or genetic PCa was estimated to be between 5 to 15% of all cases (67, 68). Racial disparities in the incidence of PCa showed that heredity was a significant factor in oncogenesis (69). Individuals sharing a common genetic background were more likely to exhibit mutations in specific genes (such as chromosome 8q24), which could contribute to an increased susceptibility to PCa (70). Next, lifestyle factors such as diet, tobacco use, and alcohol consumption could contribute to differences in PCa incidence and mortality rates among various populations (65, 71, 72). Research has shown that poor dietary habits, including high consumption of red and processed meats and low intake of fruits and vegetables, as well as smoking and heavy alcohol use, could play a role in influencing the risk of developing PCa (72). Furthermore, exposure to certain environmental factors, including chemical agents, might also contribute to the risk of developing this disease (71–73). In families affected by PCa, a combination of shared genetic predisposition, similar lifestyle factors, and potentially common environmental conditions might contribute to the occurrence of familial prostate cancer (74).

Indeed, lifestyle changes could reduce the risk of various types of cancer, including PCa. It has been reported that adopting healthy lifestyle habits could prevent approximately 30 to 50% of cancer cases (65, 71, 73, 75). Various nutrients could play a role in the pathogenesis and progression of PCa through diverse mechanisms. Vitamin D and its analogs had a potential role in preventing PCa by influencing cellular processes such as inhibiting cell multiplication and invasion, as well as modulating inflammatory signaling. Studies have revealed an association between vitamin D deficiency and an increased risk of PCa, especially among older males (42, 71). In addition, excessive intake of calcium-rich dairy products, particularly above the recommended daily intake, might be associated with an increased risk of PCa. One of reason for this association was that high calcium intake could lead to reduced serum vitamin D levels (76–79). This reduction in vitamin D levels might, in turn, contribute to an elevated risk of developing PCa. This evidences pointed to the potential impact of dietary factors and lifestyle choices to cancer prevention.

Research suggested a positive correlation between per capita animal-derived food consumption and PCa mortality (65, 75, 80). As one of animal-derived foods, milk is rich in saturated animal fats and a small amount of trans animal fats (81). Numerous components present in milk have been associated with the risk of prostate cancer. High dairy intake, particularly high consumption of high-fat dairy products, might be associated with an increased risk of PCa morbidity (82). Total dairy intake, including calcium from dairy products, had a positive relation with the risk of PCa. Low fat milk intake was related to an increased risk of non-aggressive PCa, while whole milk intake was related to an increased risk of lethal PCa (23). Research has suggested that dairy products could influence IGF levels in the blood (83), and the milk fat percentage in dairy has been associated with c-peptide levels, which were factors believed to play a role in carcinogenesis, especially in the development of aggressive forms of PCa (84). In each of the 14 individual experiments, cow’s milk stimulated the growth of LNCaP prostate cancer cells, resulting in an average growth rate increase of over 30% (85). Factors linked to the aspects of PCa promotion and progression, such as androgen signaling, increased levels of reactive oxygen species (ROS), elevated levels of prostaglandins derived from fat metabolism, heightened IGF levels, and cancer cell proliferation, were all believed to be connected with the intake of trans and saturated animal fats (71, 86). The presence of estrogen and IGF-I in milk established a connection between heightened dairy intake and the risk of prostate cancer (87). Hormonal changes, particularly related to androgen signaling, were strongly implicated in the development and progression of PCa. In some cases, alterations in the hormonal milieu, including changes in the levels of androgens such as T and dihydrotestosterone, could contribute to the development of more aggressive forms of prostate neoplasms (65, 75, 80). The changes in circulating levels of metabolic hormones and sex steroid hormones in overweight males have been linked to the development and progression of PCa (88). Hence, the hormone residues present in milk could disrupt the hormonal balance in the consumer’s body, subsequently elevating the risk of prostate cancer.

### 3.4 The mechanism by which milk consumption increased the risk of breast and prostate cancer

#### 3.4.1 The mechanism by which milk consumption increased the risk of PCa

The balance between androgens and estrogens was critically important for maintaining the proper function and integrity of the mammary gland or prostate. Disruptions in the balance and proportion of androgens and estrogens could lead to alterations in prostate function and have been associated with the development and progression of PCa. An elevated ratio of E2/T has been associated with potential implications for prostate tissue health, including the development of epithelial lesions, metaplasia, and prostatitis (89, 90). Studies have observed accelerated stromal cell proliferation and the development of metaplasia and prostatitis in hypogonadal rats with increased E2 levels (91). High E2 concentrations, particularly in the presence of normal T levels, have been associated with facilitating inflammation and stromal hyperplasia in the prostate (92). On the other hand, high androgen levels have been observed to mitigate the proinflammatory effects caused by elevated E2 concentrations, potentially exerting a protective influence on prostate health (93, 94). Studies involving nucleus basalis lesioned (NBL) rats have demonstrated that while treatment with androgens alone resulted in a PCa incidence of 35–40%, combining androgens with E2 markedly increased the incidence to 90–100% (95). Many factors, such as autoimmunity, irritants, obesogenic diets, epigenetic factors, aging, and endocrine disorders, could contribute to chronic prostate inflammation or noninfectious prostatitis. These factors might lead to an elevated ratio of E2/T, which could activate estrogenic signaling and potentially trigger noninfectious prostatitis (96–98). Adipose tissue contained aromatase and, in conditions of increased adiposity, there could be higher levels of aromatase activity. This heightened aromatase activity could lead to an increased conversion of T to E2, potentially influencing prostate health and contributing to conditions such as prostatitis and even prostate cancer in male (99). Therefore, obesity has been linked to an increased risk of various prostate conditions, including prostatitis and prostate cancer.

The presence of estrogen receptors (ERα and ERβ) and G protein-coupled ERs (GPERs) in stem cells and early progenitor cells within the normal prostate has been documented (100). These receptors played a role in mediating the cellular responses to estrogen within the prostate tissue. ERα had been associated with stimulating effect, while ERβ has been linked to inhibitory effects. GPERs had the ability to bind estrogens and might contribute to a diverse range of cellular responses to estrogen signaling, often by modulating various pathways and cellular functions (101). ERα had been associated with epithelial-mesenchymal transition and osteoblast bone formation in a mouse model of PCa (102). E2 had the similar affinity for ERα and ERβ (103). The binding of E2 to both ERα and ERβ receptors could lead to diverse cellular responses, contributing to various aspects of physiology and pathology in the context of estrogen signaling. The activation of ERα and ERβ had been associated with the enhancement of migration and invasion in DU-145 cells, as well as the promotion of invasion and anchorage-independent growth in PC-3 cells (104–106). Galectin-3 (GAL-3) and its ligands played a critical role in regulating various cellular processes, including cell proliferation, differentiation, survival and apoptosis (107). The expression of GAL-3, which was regulated by ERα and ERβ, might be involved in the transcriptional regulation of ER and direct activation of signaling cascades. The interaction between ER and some regulatory elements in the promoter domain of the human GAL-3 gene triggered genomic signaling (108). Moreover, GAL-3 interacted with nuclear factors to regulate the expression of genes associated with tumor plasticity (109). Furthermore, ERs had been shown to activate crucial signaling pathways such as the SRC/MAPK and PI3K/AKT pathways. These pathways played key roles in regulating various cellular processes, including cell proliferation and survival (110). The complex crosstalk between estrogen receptors, androgen signaling, GAL-3, and various signaling pathways indicated the multifaceted nature of the molecular and cellular mechanisms involved in the development and progression of PCa.

#### 3.4.2 The mechanism by which milk consumption increased the risk of BC

Estrogen could initiate BC by promoting the proliferation of mammary epithelial cells (111). Estroquinone, a byproduct of estrogen metabolism, has been identified as a mutagen that could bind to DNA, forming adducts and causing DNA damage (112, 113). In addition, the metabolic process of estrogen could lead to the production of ROS, which in turn could promote lipid peroxidation, resulting in the formation of lipid hydroperoxides. Both ROS and lipid hydroperoxides had the potential to cause damage to DNA and exert mutagenic effects (113). The oxidative damage induced by ROS and lipid hydroperoxides could affect DNA integrity, leading to potential mutations and contributing to cellular genomic instability. Studies have revealed that estrogen and its metabolites could induce DNA double-strand breaks (DSBs) in epithelial cells of both normal and ER-breast mammary cells (114). Therefore, estrogen had the potential to cause DNA damage directly within mammary epithelial cells, thereby increasing the risk of tumorigenesis.

Estrogen exerts its effects by binding to estrogen receptors (ER), and ERs were found to be present at high levels in tumor tissues (115). ERα was frequently detected in a significant proportion of BC, typically found in 50–80% of cases. The expression of ERα has been associated with better prognosis and lower recurrence rates in BC patients (115, 116). ERβ could be detected in breast tumors and might be associated with hormone sensitivity as well as potential implications in drug resistance (117, 118). The binding of estrogen to ER could influence cell proliferation and apoptosis in BC tissues through genomic and non-genomic pathways. Genomic actions involved the direct regulation of gene expression by activated ER, which, in turn, could impact the regulation of various cellular functions including cell proliferation and cell death. Once the ERα dimers were activated, they could translocate into the nucleus and interact with specific DNA sequences in the promoter regions of target genes. This binding allowed ERα dimers to directly regulate transcription and influence the expression of estrogen-responsive genes, thereby modulating various cellular processes (119, 120). The genomic action of ERα involved its interaction with certain transcription factors to modulate the activation or inhibition of target genes, thus affecting various cellular functions and physiological responses (115, 121). The activation of ERα through phosphorylation of kinases such as p38/MAPK/JNK, p44/42/MAPK, PI3K/Akt, and 90rsk, even in the absence of estrogen ligands, could lead to altered regulation of genes involved in cell proliferation and survival (122–124). This process has been associated with implications for endocrine resistance, particularly in the context of hormone-driven cancers such as BC.

Non-genomic pathways were characterized by rapid, non-transcriptional effects of estrogen signaling, influencing cell proliferation and apoptosis. In these pathways, ER could interact with membrane proteins located within specialized microdomains called lipid rafts, and could lead to rapid cellular responses. Mutated ER isoforms like ERα36 (125), predominantly found in lipid rafts, exhibit altered non-genomic signaling capabilities. These ERs also interacted with proteins in various kinase signaling pathways, such as PLC/PKC, Ras/Raf/MAPK, PI3K/AKT, and cAMP/PKA (126). GPERs have been found to mediate non-genomic pathways signaling pathways. As an example, GPR30, exhibited similar functions to ERα and had been found to activate the epidermal growth factor receptor (EGFR), contributing to diverse cellular responses (127, 128). EGFR was transactived by GPERs through a ligand-dependent pathway. This interaction had been associated with the stimulation of cell proliferation (127). The up-regulation of GPERs expression by E2 through the regulation of the miR-124/CD151 pathway has been linked to the acceleration of proliferation, invasion, and migration of BC cells (129). Peptide GPERs modulator like ERα17p has been shown to interact with the extracellular ligand-binding region of GPERs and induced its downregulation (130). While ERα residues might constitute an interaction platform responsible for GPERs recruitment (131). The molecular mechanisms related to non-genomic events in ER signaling were closely linked to the structural flexibility and functional properties of specific domains near the ligand-binding pocket of ERα (132). The structural characteristics of ERα played a fundamental role in dictating its functional behavior, including its binding to estrogen ligands, interaction with co-regulatory proteins, and modulation of genomic and non-genomic signaling pathways. Overall, estrogen’s influence extended across multiple signaling pathways encompassing cell proliferation, survival, DNA repair, and apoptosis, interconnecting with other oncogenic pathways to form a complex network contributing to carcinogenesis (Table 3).

**Table 3:** Potential pathways through which hormone residues in milk might elevate the risk of BC and PCa.

Pathway	Results	Sample source	References
Elevated ratio of E2/T	Prostatitis	Blood from aggressive prostate cancer, early-onset prostate cancer, and normal subjects	(88)
		serum from non-bacterial male accessory gland infection	(89)
Activation of ERα and ERβ	Promotion of invasion and anchorage-independent growth in PC-3 cells	human androgen-independent prostate cancer cell line (PC-3)	(104)
	Promotion of migration and invasion of DU-145 cells	androgen-independent DU-145 prostate cancer cells	(106)
Activation of ER, regulated GAL-3 expression	Regulate the expression of genes associated with tumor plasticity	cell lines DX3 and TXM-40; Female athymic BALB/c nude mice	(109)
Activation of ER	Activated the SRC/MAPK and PI3K/AKT pathways, cell proliferation and survival	the immortalized human breast epithelial cells MCF-10F	(110)
Promoting the proliferation of mammary epithelial cells	Increased the risk of breast cancer	Ovariectomized female rhesus monkeys (*Macacca mulatta*)	(148)
The conversion of estradiol to genotoxic metabolites in breast tissue, ER independent mechanisms	E2 influenced the development of breast cancer	castrate ERKO/Wnt mice	(110)
Estrogen metabolism produced estroquinone	Estroquinone bound to DNA causing DNA damage	Breast biopsy tissue from women diagnosed with histopathologically confirmed benign breast disease and women with breast cancer	(149)
Estrogen metabolism produced ROS, and ROS promoted lipid peroxidation to form lipid hydroperoxides	Caused DNA damage and exerted mutagenic effects		(111)
Activated ER, direct regulated gene expression	Impacted the regulation of various cellular functions	Chinese hamster ovary (CHO) cells	(119)
Activation ERα by phosphorylation of kinases	Regulated genes of cell proliferation and survival	human cervix epithelioid carcinoma HeLa cells, African green monkey kidney COS-1 cells, or rat osteosarcoma Ros cells	(122)
ER interacted with membrane proteins	Led to rapid cellular responses	human breast cancer cell lines T47D and MDA-MB-231, SKBR3 cell	(130, 131)
E2 regulated the miR-124/CD151 pathway to up-regulate GPERs expression	Accelerated proliferation, invasion, and migration of BC cells	human ER-positive breast cancer cell line, MCF-7	(130)

## 4 Appropriate milk consumptions to protect against BC and PCa

Estrogen levels have been implicated in influencing the development of cancer (133). Several studies have implicated milk consumption in potentially leading to increased concentrations of estrogen and progesterone in the blood (134). Modern high-yield, continuous commercial milk production methods could result in additional concentrations of estrogen in milk. The presence of elevated estrogen levels in commercially produced milk has been related to potential public health, including the suggestions that increased estrogen exposure through dairy consumption could had an impact on hormone-related conditions such as PCa, BC and other cancers (135). Animal-derived foods, including dairy products, were major components of the Western diet, and the increased risk of carcinogenesis might be connected with the overall carcinogenic effect of the Western diet (79).

The estrogen content in dairy products could vary and it was influenced by multiple factors. The raw milk from cows might contain natural estrogens. Estrogen levels in milk might vary due to differences in the milk production process and the types of dairy products. The processing of dairy products, including pasteurization and other treatments, might have an impact on the estrogen content of the end products. Pasteurized milk refers to milk processed using the pasteurization method, which involved high-temperature short-time treatment to kill harmful bacteria in the milk, followed by rapid cooling to preserve the nutritional components of the milk. Studies indicated that E2 levels in pasteurized milk were not substantially altered, only reducing <5% (34). Fermented milk is produced by adding appropriate probiotic bacteria to typically heat-treated animal milk, which helped convert lactose into lactic acid, resulting in extended shelf life, improved taste, and enhanced nutritional value of dairy products. The fermentation process and the product’s acidity levels did not significantly impact the estrogen content, including E2, in the final yogurt product (44). These variations in estrogen levels complicated the assessment of potential hormonal content in dairy products and their implications for human health. Moreover, the concentration of E2 was influenced by the fat content, leading to higher levels in products with higher fat content such as cream and butter. The lower hydrophobicity of E2 might contribute to its reduced levels in buttermilk in comparison to butter and even milk. The estrogen content in cream and butter was not affected by short-term product storage but was significantly reduced with longer refrigeration or freezing for 3 months, especially frozen at −18 °С, which could impact the stability of estrogen in dairy products, leading to decreased estrogen content (136). Therefore, dairy products should be appropriately consumed, especially to reduce the intake of dairy fat as a potential means to moderate the intake of sex steroid estrogens. Dietary choices, including the selection of low-fat dairy options and moderation in consumption, were key factors in promoting a healthy and balanced diet. The acceptable daily intake (ADI) of E2 was 50 ng/kg bw (136). The safe amount of milk consumption was estimated based on the estimated daily intake (EDI) of sex steroid hormones, the average body weight of the consumer, milk consumption, and sex steroid hormone levels in milk, in conjunction with the recommended ADI and the Hazard Quotient index. The recommended excess intake of total estrogen ranged from 30–50 ng/p/d, which was less than 2% of ADI (136).

In general, milk and dairy products might contain trace amounts of sex steroid hormone residues, including estrogen and progesterone. These naturally occurring hormones were present in the milk of lactating animals, and could be transferred to dairy products. These hormones might play a role in carcinogenesis and disease progression in organs such as the prostate and breast. These hormones could exert their effects through multiple molecular signaling pathways, influencing various cellular processes that were relevant to cancer development and progression. The stability of sex steroid hormones, particularly estradiol (E2), in dairy products and the potential risk of E2 residues through the intake of milk and dairy products were important considerations. Consumers might have concerns about the presence of such residues and their potential health impacts. And, various factors could influence hormone levels in dairy products. Progesterone residues in milk and dairy products were generally considered to pose a very small risk when consumed. Progesterone might not be a significant factor in terms of potential health concerns for consumers. Meanwhile, the sex steroid hormone content of milk and dairy products should be regularly monitored to ensure consumer safety and to guide evidence-based dietary recommendations. Therefore, controlling the intake of milk and dairy appropriately and making informed choices regarding milk products could contribute to reducing potential health risks associated with sex steroid hormone content, including concerns related to PCa and BC.
